# Relative importance of four functional measures as predictors of 15-year mortality in the older Dutch population

**DOI:** 10.1186/s12877-019-1092-4

**Published:** 2019-03-25

**Authors:** Elisabeth M. W. Eekhoff, Natasja M. van Schoor, Joseph S. Biedermann, Mirjam M. Oosterwerff, Renate de Jongh, Nathalie Bravenboer, Mireille N. M. van Poppel, Dorly J. H. Deeg

**Affiliations:** 1 Department of Internal Medicine, Section Endocrinology, Amsterdam University Medical Centers location VU University Medical Center, De Boelelaan 1117, 1081HV, Amsterdam, The Netherlands; 2 Department of Epidemiology and Biostatistics/LASA, Amsterdam Public Health Research Institute, Amsterdam University Medical Centers location VU University Medical Center, Van der Boechorststraat 7, 1081 BT Amsterdam, The Netherlands; 3 Department of Internal Medicine, Reinier de Graaf Gasthuis, Reinier de Graafweg 5, 2625 AD Delft, The Netherlands; 4 Department of Internal Medicine, Catharina Hospital, Michelangelolaan 2, 5623 EJ Eindhoven, The Netherlands; 5 Department of Clinical Chemistry, Amsterdam Movement Sciences, Amsterdam University Medical Centers, Vrije Universiteit, De Boelelaan 1117, 1081HV Amsterdam, The Netherlands; 6 Department of Public and Occupational Health, Amsterdam Public Health Research Institute, Amsterdam University Medical Centers location VU University Medical Center, Amsterdam, The Netherlands; 7 Institute of Sport Science, University of Graz, Graz, Austria

**Keywords:** Lower-body performance, Handgrip strength, Lung function, Functional limitations, Mortality, LASA

## Abstract

**Background:**

Decreased physical function is known to raise mortality risk. Little is known about how different physical function measures compare in predicting mortality risk in older men and women. The objective of this study was to compare four, objective and self-reported, physical function measures in predicting 15-year mortality risk in older men and women.

**Methods:**

Data were used from the Longitudinal Aging Study Amsterdam (LASA), an ongoing cohort study in a population-based sample of the older Dutch population, sampled from municipal records. The 1995–96 cycle, including 727 men and 778 women aged 65–88 years, was considered as the baseline. Mortality was followed up through September 1, 2011. Physical function measures were: lower-body performance (chair stands test, walk test and tandem stand); handgrip strength (grip strength dynamometer); lung function (peak expiratory flow rate); functional limitations (self-report of difficulties in performing six activities of daily living). Cox proportional hazard models were used to determine the predictive value of each physical function measure for 15-year mortality risk, adjusted for demographic, lifestyle and health variables as potential confounders.

**Results:**

1031 participants (68.5%) had died. After adjustments for confounders, in models assessing single functional measures, peak flow was the strongest predictor of all-cause mortality in men (HR 1.76, CI 1.38–2.26, CI) and lower-body performance in women (HR 1.97,CI 1.40–2.76, CI). In a model including all four functional measures only peak flow was statistically significant in predicting mortality in both genders (men HR 1.54,CI 1.18–2.01 and women HR 1.45,CI 1.08–1.94). In women, lower-body performance (HR 1.66, CI 1.15–2.41) followed by grip strength (HR 1.38, CI 1.02–1.89), and in men, functional limitations (HR 1.43, CI 1.14–1.8) were the other significant predictors of all-cause mortality.

**Conclusion:**

Both objective and self-reported measures of physical functioning predicted all-cause mortality in a representative sample of the older Dutch population to different extents in men and women. Peak flow contributed important unique predictive value for mortality in both men and women. In women, however, lower-body performance tests had better predictive ability. A second-best predictor in men was self-reported functional limitations. Peak flow, and possibly one of the other measures, may be used in clinical practice for assessment in the context of time constraints.

## Background

The ageing process is accompanied by the loss of physical function. Physical function is the common term used to describe the combination of endurance, muscle power, balance and coordination which capacity is related to exercise, health status, and genetic and environmental factors. The maximal physical function decreases gradually due to ageing from early midlife.

Physical function can be assessed by many different tests, among which objective tests, such as lower-body performance, hand grip strength and peak flow, and self-reported measures, such as reports of experiencing difficulty in performing activities of daily living (i.e. self-reported functional limitations). Several studies in older persons have linked poorer physical function with negative health outcomes. For example, lower-body performance tests have been associated with hospitalization and increased mortality risk [1–6], and poorer hand grip strength have been associated with frailty and an increased mortality risk [7–10]. Furthermore, it has been shown that peak expiratory flow rate is a strong predictor for mortality risk [11]. Also self-reported measures, such as functional limitations have been associated with an increased mortality risk [12–14].

Although many studies examined the predictive value of physical function measures on mortality risk, little is known about how these different physical function measures compare in predicting mortality risk. Two recent studies in older persons compared the ability of four and seven different objective physical function measures in predicting mortality [15, 16], i.e., the Short Lower-body performance Battery (SPPB), the 4-m walk test (4-mWT), the 6-min walk test (6-MWT) and hand grip strength in one study [15] and similar but with chair stands time, leg extension and flexion on top in the other study [16]. In the multivariable analyses, only SPPB and 6-MWT in both studies but also 4-mWT in the second study, showed a significant inverse association with 7- and 4.4-year mortality risk for both genders respectively [15, 16]. One cross-sectional study showed that four physical function tests, including peak flow as well as hand grip strength, leg strength and physical activity, were all associated with impaired physical ability and disability in activities of daily living in older persons (ADL) [17].

Although gender was a significant predictor in most of these studies, men and women were examined separately only in the study by Veronese and colleagues [16]. Stratification by gender may be of importance if there is an indication for gender differences: indeed, pronounced gender influences have been observed regarding the predictive values of lower muscle strength, grip strength and functional disabilities for increased mortality risk in older persons [5–7, 9, 11–14, 16–18]. The study by Veronese and colleagues showed gender differences in the prediction of mortality risks of physical function measures specifically because of the influence of malnutrition in women over a period of 4 .4years [16].

Of the two previous studies that assessed the association between self-reported measure of physical function and mortality risk only one study [5] showed a difference between sexes [12].

For screening and in clinical diagnostic and therapeutic decision making it is important to know which of the different physical function measures are the best in predicting mortality risk. The current study is the first study where a comparison is made among four tests including both objective (lower-body performance tests, grip strength, peak flow measured by an observer) and subjective tests (self-reported physical function measures, i.e., functional limitations) in one sample of an older community dwelling population to determine the optimal test for each gender in predicting mortality over a period of 15 years.

## Methods

### Study sample

Data were used from the Longitudinal aging Amsterdam (LASA), an ongoing interdisciplinary cohort study on predictors and consequences of changes in physical, cognitive, emotional and social functioning in the older Dutch population [19, 20]. The LASA study started in 1992 with a random sample of older men and women from municipal records, stratified for age, gender, grade of urbanization and expected 5-yr mortality, drawn from the population registers of 11 municipalities in three regions of the Netherlands (Amsterdam, Zwolle and Oss, and vicinity). The baseline examination in 1992/1993 included 3107 predominantly Caucasian (> 99%) respondents. The sample for the current study consisted of participants aged 65 through 88 years as of January 1, 1996, who took part in the main and medical interview of the second LASA cycle (1995/1996) and from whom data were available on lower-body performance, handgrip strength, peak flow, functional limitations, and mortality status during 15 years of follow-up (*N* = 1505*;* 727 men and 778 women). In total, 695 men and 734 women had data on lower-body performance; 727 men and 778 women had data on hand grip strength, 722 men and 751 women on peak flow, and 717 men and 760 women on functional limitations.

The exact number available for analysis further depended on the covariables included in the model. All participants signed for informed consent and the Ethical Review Board of the VU medical center approved the study.

### Lower-body performance

Three standardized performance tests were used to assess lower-body performance, i.e., the chair stands test, primarily measuring proximal leg strength, the walk test, primarily measuring a combination of proximal leg strength, coordination and balance, and the tandem stands test, primarily measuring balance. During the chair stands test, participants were asked to fold their arms across their chest and to stand up and sit down from a sitting position five consecutive times as quickly as possible. Scores ranged from one to four corresponding to the quartiles of time required in the total population. These were recoded such that 4 = best and 1 = worst performance. A score of 0 was assigned if the participant was unable to perform the test [21]. During the walk test, participants were asked to walk 3 m along a line, turn around and walk back as quickly as possible without running. Time to complete the test was recorded and scores were assigned in a similar way as the chair stands test. During the tandem stand test, the participant was asked to stand with one foot in front of the other, with their heel against their toe, for 10 s. A score of 2 points was assigned if the participant was able to hold position for 3–9 s; 4 points if able to hold on for 10 s or longer. The score of 0 was assigned if participant held the position for less than 3 s or was unable to perform the test. A lower-body performance score (range 0–12) was computed by summing the scores of the three individual performance tests with a higher score reflecting good lower-body performance.

### Handgrip strength

Handgrip strength was measured using a grip strength dynamometer (Takei TKK 5001, Takei Scientific Instruments Co. Ltd., Tokyo, Japan) [22]. Measurements were done in duplicate for both hands and the highest measured result of each hand was used to calculate the average grip strength. Grip strength was recorded to the nearest 1 kg. The dynamometer was adjusted for the hand size of the participant.

### Lung function

Lung function was assessed by the peak expiratory flow rate using the Mini-Wright peak flow meter. Peak expiratory flow rate is defined as a person’s maximum speed of expiration. For the measurements, the subjects were instructed to take a maximum inspiration and to breathe out with maximum effort into the peak flow meter. The highest score of three measurements in milliliters (ml) was used [17].

### Functional limitations

Participants were asked to which degree they experienced difficulties in performing the following activities of daily living: going up and down the stairs, getting (un-) dressed, sitting down and rising from a chair, cutting one’s own toenails, walking 400 m, and using own or public transportation. [22] Response categories included “yes, without difficulty; yes, with some difficulty; yes, with much difficulty; only with help; no, I cannot” with corresponding scores ranging from 1 to 5. A functional limitation score (range 6–30) was computed by summing the scores of the individual questions. A higher score indicates more difficulties.

### 15-year mortality follow-up

Vital status and date of death of participants were acquired through linkage with municipal registries through September 1, 2011. Follow-up data were complete for 1505 out of 1509 persons.

### Potential confounders

Potential confounders derived from previous studies included demographic variables (age, gender, educational level, urbanization grade, partner status), lifestyle variables (smoking, alcohol use), and health status variables (number of chronic diseases, medication use, body mass index, cognitive impairment) [23]. Data regarding age, gender and urbanization grade were extracted from population registries at baseline. Education level in years was determined by the respondent’s highest completed level of education. Partner status was assessed by self-report. Smoking was assessed by self-report and categorized into never, former, current smoker. Alcohol consumption (does not drink, light, moderate, and excessive drinking) was assessed by the Garretsen Index [24]. Body height was measured using a stadiometer. Body weight was measured without clothes and shoes using a calibrated bathroom balance scale. Body mass index (BMI) was calculated as body weight in kg divided by height in meters squared. Presence and number of 7 major chronic diseases was assessed by self- report: chronic obstructive pulmonary disease (COPD), cardiac disease, vascular disease, stroke, diabetes mellitus, malignant neoplasms and joint disorders (osteoarthritis, rheumatoid arthritis). Medication use was assessed by asking the participants to show their medication containers to the interviewers. The number of medications was used in the analyses. Cognitive impairment was assessed with the Mini-Mental State Examination [25]. A score below 24 indicates the presence of a cognitive impairment [26].

#### Statistical analysis

First, survivors were compared with non-survivors using independent samples T-Test for normally distributed variables; Mann-Whitney U test for skewed variables, and Pearson Chi-square test for categorical variables. The data distribution test used was the commonly used Kolmogorov-Smirnov test.

Second, sex-specific correlation coefficients were calculated between the four physical function measures to assess their interrelation. Both the unadjusted and the age-adjusted coefficients were calculated. Third, the association between the sex-specific tertiles of the physical function measures and mortality was examined using Cox Proportional Hazards Models. Each measure was categorized in tertiles, first, to account for non-linear associations, and second, to be able to compare the hazard ratios across the measures. The tertiles were obtained by considering the frequency distribution of each physical function measure for men and women separately.

Gender was examined as a potential effect modifier by adding an interaction term to the univariable model. In case of one or more statistically significant interaction effects (*p* < 0.1), all further analyses were stratified by sex. Then, potential confounders associated with both the predictor and the outcome (p < 0.1), were added to the model. Two models are reported, one model adjusted for age and gender (if no interaction was present) and a second model adjusted for all relevant confounders. Finally, the relative importance of the four physical function measures was examined by including all four physical function measures into the adjusted model. This was done on the provision that the age-adjusted intercorrelations between the four measures did not exceed 0.60. All analyses were conducted using SPSS software version 21.0 (SPSS Inc., Chicago, IL).

## Results

### Descriptives

The main characteristics of the study sample (*N* = 1505) are presented in Table 1 according to mortality status on September 1st, 2011. The average age at baseline was 76.0 years (SD 6.6) and females comprised 51.6% of the sample. Through September 1st, 2011, 1031 participants died (68,5%). The mean follow-up time was 15.4 years (±0.3) for survivors, and 7.2 (±4.3) for the deceased.

**Table 1:** Baseline characteristics of the study sample stratified by mortality status at 1 September 2011

	Total	Survivor	Non-survivor	P
No. of subjects	1505	474	1031	
Age	76.0 ± 6.6	71.2 ± 4.7	78.1 ± 6.3	< 0.001
Gender				< 0.001
male	727	177 (24.3)	550 (75.7)	
female	778	297 (38.2)	481 (61.8)	
Urbanicity level				0.003
Not (< 500 addresses/km^2^)	280	96 (34.3)	184 (65.7)	
Little (500–1000)	325	118 (36.3)	207 (63.7)	
Somewhat (1000-1500)	154	55 (35.7)	99 (64.3)	
Highly (1500–2500)	367	104 (28.3)	263 (71.7)	
Very highly (> 2500)	378	101 (26.7)	277 (73.3)	
Education level				
- Years	8.9 ± 3.3	9.1 ± 3.2	8.8 ± 3.4	0.038
Living with partner				
- Yes	809	299 (37.0)	510 (63.0)	
- No	696	175 (25.1)	521 (74.9)	< 0.001
Smoking				< 0.001
- Never	537	203 (37.8)	334 (62.2)	
- Former	678	218 (32.2)	468 (67.8)	
- Current	288	53 (18.4)	235 (81.6)	
Alcohol use				0.004
- None	386	96 (24.9)	290 (75.1)	
- Light	746	264 (35.4)	482 (64.6)	
- Moderate	283	89 (31.4)	194 (68.6)	
- (Very) Excessive	87	25 (28.7)	62 (71.3)	
BMI				0.011
- Underweight (< 20)	57	7 (12.3)	50 (87.7)	
- Normal (20–24)	451	154 (34.1)	297 (65.9)	
- Overweight (25–29)	662	214 (32.3)	448 (67.7)	
- Obese (> 30)	307	97 (31.6)	210 (68.1)	
No. of chronic diseases	1.7 ± 1.3	1.5 ± 1.1	1.9 ± 1.3	< 0.001
MMSE score				< 0.001
≤23	208	12 (5.8)	196 (94.2)	
≥24	1293	462 (35.7)	831 (64.3)	
Lower-body performance score (0–12)	7.1 ± 3.3	8.6 ± 2.5	6.4 ± 3.4	< 0.001
Walk test (0–4)	2.4 ± 1.2	2.9 ± 1.1	2.2 ± 1.2	< 0.001
Chair stand test (0–4)	1.9 ± 0.8	2.1 ± 0.8	1.8 ± 0.8	< 0.001
Tandem stand (0–4)	2.4 ± 0.9	2.7 ± 0.6	2.2 ± 0.9	< 0.001
Handgrip strength (kg)	29.0 ± 10.3	30.5 ± 9.8	28.3 ± 10.4	< 0.001
Peak flow (60-780 ml)	373 ± 126	417 ± 117	353 ± 125	< 0.001
Functional limitation score (6–30) ^a^	26.3 ± 5.1	28.2 ± 3.2	25.5 ± 5.6	< 0.001

Continuous variables are expressed as mean (S.D.) for normally distributed variables or median (interquartile range) for skewed variables^a^. Categorical variables are listed in numbers (%).X^2^ is used for categorical variables, independent T-test for continuous variables with a normal distribution and Mann-Whitney-U for skewed variables

Participants who died during follow-up differed from those who survived on all covariates: they were older, more often male and living in highly urban areas, had fewer years of education, fewer lived with a partner, they were more often current smoker, more often did not consume alcohol or did so excessively, more often were underweight, and scored worse on all health indicators. Likewise, all measures of physical functioning, lower-body performance (and its components), handgrip strength, peak flow, and self-reported functional limitations, were significantly lower (*P* < 0.001) at baseline in those who died during follow-up compared to those who survived.

The partial correlations among the four measures of physical functioning, controlling for age, ranged from 0.24 (self-reported functional limitations and peak flow) to 0.58 (performance tests and self-reported functional limitations) for men, and from 0.18 (self-reported functional limitations and peak flow) to 0.58 (performance tests and self-reported functional limitations) for women (Table 2).

**Table 2:** Sex-specific correlations^a^ among the four function measures: zero-order (before slash) and partial correlation controlling for age (after slash), respectively

	Lower-body performance	Grip strength	Peak flow
*Men*			
Lower-body performance	–		
Grip strength	0.50 / 0.34 *N* = 694	–	
Peak flow	0.38 / 0.24 *N* = 690	0.39 / 0.26 *N* = 721	–
Functional limitations	0.66 / 0.58 *N* = 684	0.45 / 0.33 *N* = 716	0.34 / 0.24 *N* = 711
*Women*			
Lower-body performance	–		
Grip strength	0.47 / 0.26 *N* = 735	–	
Peak flow	0.38 / 0.20 N = 711	0.42 / 0.27 *N* = 753	–
Functional limitations	0.70 / 0.58 N = 721	0.47 / 0.30 *N* = 761	0.34 / 0.18 *N* = 736

^a^Each correlation calculated using the maximum number of cases available

### Gender differences in the relationship between sex-specific tertiles of the four functional measures and mortality

Significant gender interaction was observed in the association between the sex-specific tertiles of lower-body performance and mortality (interaction term *P* = 0.003). No significant gender interaction was present for grip strength, functional limitation score, and peak flow (interaction terms *P* > 0.10). To improve comparability between findings, all further analyses were stratified for sex.

### Sex-specific tertiles of the functional measures and mortality

**Fig. 1:**
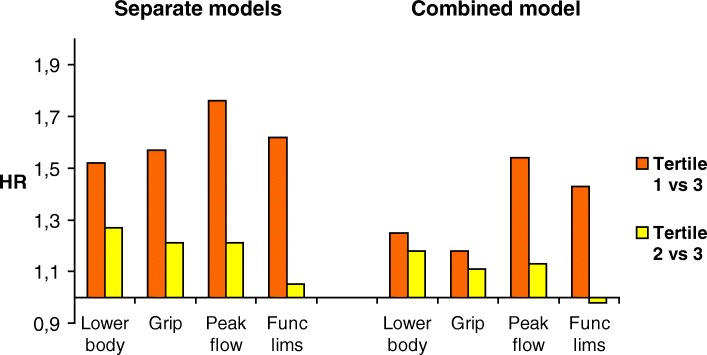
Hazard ratios for mortality of sex-specific tertiles of physical function measures, men. Notes: HR = Hazard Ratio; Lower body = Lower body performance; Grip = Grip strength; Peak flow = Peak expiratory flow; Func lims = Functional limitations; Separate model = Separately for each test; Combined model = Contribution if all four tests combined

**Fig. 2:**
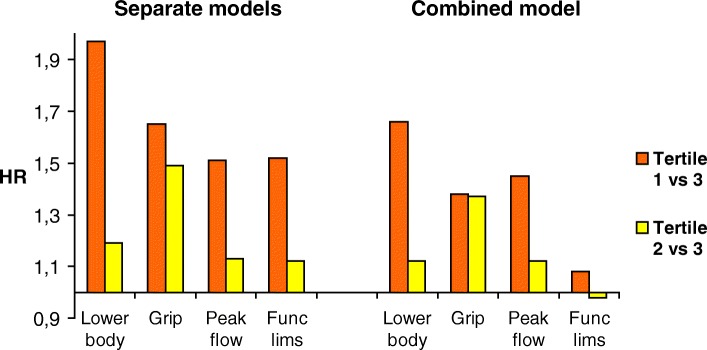
Hazard ratios for mortality of sex-specific tertiles of physical function measures, women. Notes: HR = Hazard Ratio; Lower body = Lower body performance; Grip = Grip strength; Peak flow = Peak expiratory flow; Func lims = Functional limitations; Separate model = Separately for each test; Combined model = Contribution if all four tests combined

In the crude analyses, all measures of physical function were significantly associated with mortality for both sexes (Table 3, Figs. 1 and 2, left panels). Corrected for all relevant confounders, mortality risk was more than one and a half to nearly twice as high for women in the lowest tertiles of handgrip strength (HR 1.65, CI 1.25–2.16) and of lower-body performance (R 1.97, CI 1.40–2.76) in comparison to women in the highest tertiles. Mortality risk of men in the lowest tertiles of functional limitations (HR 1.62, CI 1.31–2.0) and of peak flow (HR 1.76, CI 1.38–2.26) was significantly higher compared to their peers in the corresponding highest tertiles.

**Table 3:** Hazard ratios for mortality of sex-specific tertiles of physical function measures, separate models for each functional measure

	Model 1 HR 95% CI	Model 2 HR 95% CI	
Lower-body performance (range)			
*Men*			
Tertile 1 (0–7)	1.70 (1.36–2.14)	1.52 (1.20–1.92)	
Tertile 2 (8–9)	1.30 (1.02–1.65)	1.27 (0.99–1.61)	
Tertile 3 (10–12)	Reference	Reference	
*Women*			
Tertile 1 (0–6)	2.06 (1.50–2.83)	1.97 (1.40–2.76)	
Tertile 2 (7–8)	1.23 (0.90–1.70)	1.19 (0.86–1.65)	
Tertile 3 (9–12)	Reference	Reference	
Grip strength			
*Men*			
Tertile 1 (10–33)	1.66 (1.32–2.10)	1.57 (1.23–1.99)	
Tertile 2 (34–40)	1.17 (0.94–1.46)	1.21 (0.97–1.51)	
Tertile 3 (41–69)	Reference	Reference	
*Women*			
Tertile 1 (16–19)	1.83 (1.41–2.38)	1.65 (1.25–2.16)	
Tertile 2 (20–23)	1.50 (1.18–1.92)	1.49 (1.16–1.91)	
Tertile 3 (24–39)	Reference	Reference	
Peak flow			
*Men*			
Tertile 1 (60–365)	2.21 (1.75–2.80)	1.76 (1.38–2.26)	
Tertile 2 (370–495)	1.37 (1.09–1.73)	1.21 (0.95–1.53)	
Tertile 3 (500–780)	Reference	Reference	
*Women*			
Tertile 1 (60–285)	1.79 (1.39–2.32)	1.51 (1.16–1.98)	
Tertile 2 (290–365)	1.25 (0.96–1.62)	1.13 (0.87–1.46)	
Tertile 3 (370–700)	Reference	Reference	
Functional limitations			
*Men*			
Tertile 1 (10–28)	1.93 (1.59–2.35)	1.62 (1.31–2.00)	
Tertile 2 (29)	1.12 (0.87–1.44)	1.05 (0.81–1.35)	
Tertile 3 (30)	Reference	Reference	
*Women*			
Tertile 1 (6–25)	1.62 (1.26–2.08)	1.52 (1.13–2.04)	
Tertile 2 (26–29)	1.10 (0.85–1.44)	1.12 (0.85–1.49)	
Tertile 3 (30)	Reference	Reference	

To facilitate comparability, the models for all function measures and each sex include the same covariates:

Model 1: Adjusted for age

Model 2: Adjusted for age, no. of chronic diseases, cognitive impairment, body mass index, smoking, alcohol use

### Relative importance of the four functional measures

The models including all four measures (Table 4) showed a marked gender difference in predictive ability with the strongest combination of measures of peak flow (HR 1.54, CI 1.18–2.01) and self-reported functional limitations (HR 1.43, CI 1.14–1.8) in men and peak flow (HR 1.45, CI 1.08–1.93) and lower-body performance (HR 1.66, CI 1.15–2.41) and to a lesser extent grip strength (HR 1.38, CI 1.02–1.89) in women. In men, the hazard ratios of lower-body performance and grip strength were no longer significant. In women, functional limitations were no longer significant.

**Table 4:** Hazard ratios for mortality of sex-specific tertiles of physical function measures, for each gender one combined model

	Men, Model 1 HR 95% CI	Men, Model 2 HR 95% CI	Women, Model 1 HR 95% CI	Women, Model 2 HR 95% CI
*Performance tests (range)*				
Tertile 1 (Men 0-7; Women 0-6)	1.29 (1.01–1.66)	1.25 (0.97–1.62)	1.58 (1.10–2.27)	1.66 (1.15–2.41)
Tertile 2 (Men 8-9; Women 7-8)	1.18 (0.92–1.51)	1.18 (0.92–1.51)	1.11 (0.79–1.56)	1.12 (0.79–1.57)
Tertile 3 (Men 10-12; Women 9-12)	Reference	Reference	Reference	Reference
*Grip strength*				
Tertile 1 (Men 10-33; Women 6-19)	1.16 (0.90–1.50)	1.18 (0.90–1.53)	1.44 (1.07–1.93)	1.38 (1.02–1.89)
Tertile 2 (Men 34-40; Women 20-23)	1.08 (0.86–1.35)	1.11 (0.88–1.40)	1.30 (1.00–1.69)	1.37 (1.05–1.80)
Tertile 3 (Men 41-69; Women 24-39)	Reference	Reference	Reference	Reference
*Peak flow*				
Tertile 1 (Men 60-365; Women 60-285)	1.84 (1.43–2.38)	1.54 (1.18–2.01)	1.62 (1.23–2.14)	1.45 (1.08–1.93)
Tertile 2 (Men 370-495; Women 290-365)	1.26 (0.99–1.60)	1.13 (0.88–1.44)	1.22 (0.92–1.60)	1.12 (0.85–1.49)
Tertile 3 (Men 500-780; Women 370-700)	Reference	Reference	Reference	Reference
*Functional limitations*				
Tertile 1 (Men 10-28; Women 6-25)	1.64 (1.33–2.03)	1.43 (1.14–1.80)	1.07 (0.79–1.44)	1.08 (0.77–1.52)
Tertile 2 (Men 29; Women 26-29)	1.04 (0.80–1.34)	0.98 (0.76–1.28)	0.96 (0.73–1.28)	0.98 (0.72–1.33)
Tertile 3 (Men 30; Women 30)	Reference	Reference	Reference	Reference

Model 1: Adjusted for age and the other functional measures

Model 2: Adjusted for age, the other functional measures, no. of chronic diseases, cognitive impairment, body mass index, smoking, alcohol use

## Discussion

In this large population-based cohort study, the predictive value of four measures of physical functioning for all-cause mortality risk in older Dutch men and women was assessed, after correction for relevant confounders. The findings demonstrate that although each objective muscle strength measure (lower-body performance, handgrip strength), objective lung function (peak flow), as well as subjective self-reported functional limitations can predict mortality in older people, remarkable differences exist between the strength of the associations for gender and mortality outcome. While decreased lower-body performance was associated with the highest increase of mortality risk in older women, strikingly, in older men lower peak flow showed the highest risk. In a search for the measures with the highest predictive ability, the model including all four measures showed a dominance of functional limitations and peak flow, which were the only two remaining significant predictors of mortality in men. In contrast, in the combined model for women, functional limitations were no longer significant. In women, the lower-body performance and, with a weaker predictive ability, peak flow and grip strength remained as significant predictors.

Peak flow appeared to be the most unique and independent predictor. In both men and women peak flow was least correlated with all other measures while lower-body performance and functional limitations were the highest correlated. Thus, peak flow adds predictive value over the other functional measures.

Adding all confounders to the separate analysis (model 2) reduced the strength of all associations compared to adjusting for age alone (model1). Adding all confounders to the combined tests analysis reduced the importance of peak flow test in both sexes, of functional limitations in men and turned physical performance in men non-significant.

To our knowledge, this is the first study that compares objective measures of physical functioning, including peak flow, and self-reported measures in their predictive value for 15-year mortality risk in a large representative sample of Dutch elderly between men and women. While prior studies also found gender differences in the relationship between mortality risk and handgrip strength [8, 9], self-reported disability in ADL [12] and functional performance [16], we are the first to show that when addressing all four tests the difference in predictive strength for mortality between men and women becomes more clearly apparent.

Our findings support prior observations that objectively measured performance-based tests are better predictors of all-cause mortality than self-reported functional limitations in older community dwelling women [5, 27]. This may tie in with the ‘gender-longevity’ paradox: Although older women have more disabilities they survive longer than men after a reported onset of disability. The test is easy to perform and has often been used in studies. Paradoxically, but in line with previous studies we show that functional limitations are a strong predictor of mortality in men [28]. It might be speculated that women can cope better with disability in daily living.

A few studies have examined the effect of gender on the association between grip strength and mortality with variable findings [6, 8, 15, 16, 18, 29–31]. Among them muscle strength was not predictive of mortality in women in four studies [8, 15, 16, 30], while four other studies [6, 18, 29, 31] reported an increased risk of mortality for women in the lowest quartile or tertile of grip strength. These last results are in line with our observations. The underlying factors that might relate the decline in muscle power to mortality are yet unclear, but seems to be multifactorial including a chronic low-grade inflammation [18].

In this study peak flow was among the strongest mortality predictors in both men and women. This association has been found before, but is not yet understood [11]. One possible explanation [32] is that peak flow is a measure of physical fitness in general. During aging, physiological and structural changes lead to more rigid and less expansible respiratory systems with a declined strength of the diaphragm, compliance of the lungs and inspiratory and expiratory intercostal muscle capacity [33]. Small changes might cause older people to be less physically active, which may have a high impact on physical fitness [34].

Our findings indicate that the use of one measure, i.e. peak flow, possibly combined with one of the other more gender specific measures may suffice to assess physical condition and subsequently mortality risk. Our work also shows the usefulness of separate analysis of both sexes. However, if one should consider only one test, the peak flow test could be considered as the best option for both genders when assessed in an older community dwelling population.

Strengths of the present study include the use of a large population-based, well-characterized study sample, the comparison between four measures separately and combined per gender, the long follow-up period and the examination of and correction for effect modification and various confounders. One of the limitations of our study is that we did not differentiate between cause-specific mortality. Also, our study did not include peak flow tests by spirometry. However, peak expiratory flow rate had been demonstrated to be a reliable predictor of lung function. Furthermore, since participants of the LASA-cohort were predominantly Caucasian, generalization of our findings to non-Caucasian older people needs further investigation. Future studies have to determine whether intervention programs aiming to increase lower-body performance, muscle strength, functional independence and peak flow decrease mortality risk of older people in low performance groups, in order to prove causality.

In addition, from our findings a new study might be developed to design a practical scoring system to predict mortality.

## Conclusions

This study investigated the predictive strength and gender differences in predictive strength of four measures of physical functioning for mortality in older men and women. In models including only one functional measure, corrected for all relevant confounders peak flow was the best predictor of mortality in older men and lower-body performance in women. In the relative contribution model, peak flow and functional limitations were the strongest predictors of mortality in older men. In contrast, two objective measures, peak flow and lower-body performance, were better predictors of mortality in older women.

## Abbreviations

4-mWT: 4-m walk test
6-MWT: the 6-min walk test
ADL: Activity of daily living
BMI: Body mass index
COPD: Chronic obstructive pulmonary disease
LASA: Longitudinal aging Amsterdam
SPPB: Short Lower-body performance Battery
